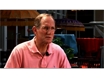# The 2009 Lindau Nobel Laureate Meeting: Peter Agre, Chemistry 2003

**DOI:** 10.3791/1565

**Published:** 2009-12-09

**Authors:** Peter Agre

## Abstract

Peter Agre, born in 1949 in Northfield Minnesota, shared the 2003 Nobel Prize in Chemistry with Roderick MacKinnon for his discovery of aquaporins, the channel proteins that allow water to cross the cell membrane.

Agre's interest medicine was inspired by the humanitarian efforts of the Medical Missionary program run by the Norwegians of his home community in Minnesota. Hoping to provide new treatments for diseases affecting the poor, he joined a cholera laboratory during medical school at Johns Hopkins. He found that he enjoyed biomedical research, and continued his laboratory studies for an additional year after medical school.

Agre completed his clinical training at Case Western Hospitals of Cleveland and the University of North Carolina, and returned to Johns Hopkins in 1981. There, his serendipitous discovery of aquaporins was made while pursuing the identity of the Rhesus (Rh) antigen.

For a century, physiologists and biophysicists had been trying to understand the mechanism by which fluid passed across the cell's plasma membrane. Biophysical evidence indicated a limit to passive diffusion of water, suggesting the existence of another mechanism for water transport across the membrane. The putative "water channel," however, could not be identified.

In 1988, while attempting to purify the 30kDa Rh protein, Agre and colleagues began investigating a 28 kDa contaminant that they believed to be a proteolytic fragment of the Rh protein. Subsequent studies over the next 3-4 years revealed that the contaminant was a membrane-spanning oligomeric protein, unrelated to the Rh antigen, and that it was highly abundant in renal tubules and red blood cells. Still, they could not assign a function to it.

The breakthrough came following a visit with his friend and former mentor John Parker. After Agre described the properties of the mysterious 28 kDa protein, Parker suggested that it might be the long-sought-after water channel. Agre and colleagues tested this idea by expressing the protein in *Xenopus* oocytes, which typically have low water permeability. When the test oocytes were placed in a hypotonic solution, they swelled and exploded, thus revealing the function of the unknown protein as a water channel, which they named aquaporin.

The Nobel Prize enabled Agre to take his research and scientific interests in new directions. He felt that over the years his work had continually taken him further from his original interests in third-world diseases, so he shifted his focus back in that direction. He now serves as the director of the Malaria institute at Johns Hopkins where he has applied his knowledge to the study of the malarial parasite and the *Anopheles *mosquito, which both express aquaporins. In addition, since winning the Nobel Prize, he has enjoyed increased opportunities for bringing science to the public and for "encouraging young people to go into science."

**Figure:**